# The effectiveness and usability of online, group-based interventions for people with severe obesity: a systematic review and meta-analysis

**DOI:** 10.1038/s41366-024-01669-2

**Published:** 2024-11-19

**Authors:** Madison Milne-Ives, Lorna Burns, Dawn Swancutt, Raff Calitri, Ananya Ananthakrishnan, Helene Davis, Jonathan Pinkney, Mark Tarrant, Edward Meinert

**Affiliations:** 1 Translational and Clinical Research Institute, Newcastle University, Newcastle upon Tyne, UK; 2 Centre for Health Technology, School of Nursing and Midwifery, University of Plymouth, Plymouth, PL4 6DN UK; 3 Peninsula Dental School, Faculty of Health, University of Plymouth, Plymouth, PL4 6DN UK; 4 Peninsula Medical School, Faculty of Health, University of Plymouth, Plymouth, PL4 6DN UK; 5 Department of Health and Community Sciences, University of Exeter, Exeter, EX1 2 LU UK; 6 Department of Clinical and Biomedical Sciences, University of Exeter Medical School, St Luke’s Campus, Exeter, EX1 2LU UK; 7 School of Psychology, Faculty of Health, University of Plymouth, Portland Square, Plymouth, PL4 8AA UK; 8 Department of Primary Care and Public Health, School of Public Health, Imperial College London, London, W6 8RP UK

**Keywords:** Weight management, Therapeutics

## Abstract

Long wait times, limited resources, and a lack of local options mean that many people with severe obesity cannot access treatment. Face-to-face group-based interventions have been found effective and can treat multiple people simultaneously, but are limited by service capacity. Digital group interventions could reduce wait times, but research on their effectiveness is limited. This systematic review aimed to examine the literature about online group-based interventions for adults with severe obesity (BMI ≥ 35 kg/m^2^). The review followed the PRISMA and PICOS frameworks. MEDLINE, Embase, CINAHL, Web of Science, and Cochrane Central Register of Controlled Trials were searched. Two authors independently screened articles. Data extraction, analysis, and quality assessment (using RoB2 and MMAT) was shared between two authors. A meta-analysis was conducted on eligible studies; other results were descriptively analysed. 20 papers reporting on 15 studies were included. Most studies reported some evidence of weight loss, but evidence of weight-related behaviour change was mixed. A meta-analysis on four studies indicated that online, group-based interventions had a statistically significant impact on weight loss (*p* = 0.001; 95% CI −0.69 to −0.17) with a small-to-moderate effect size, compared to waitlist or standard care conditions. Online interventions were considered more convenient but lack of familiarity with the group or counsellor, accessibility issues, and time constraints hindered engagement. Technical support, incentives, and interactive forums to improve group cohesion could mitigate these barriers. The findings suggested that online, group-based interventions are feasible and potentially beneficial, but barriers such as internet accessibility, digital literacy, and unfamiliarity with group members need to be mitigated. Key recommendations to improve experience and impact include providing instructions and run-throughs, building group cohesion, and providing session and additional content throughout the intervention. Future studies should focus on the influence of specific intervention characteristics and investigate the effect of these interventions compared to face-to-face interventions. Registration: National Institute for Health Research, PROSPERO CRD42021227101; https://www.crd.york.ac.uk/prospero/display_record.php?ID=CRD42021227101.

## Introduction

Obesity is a significant public health problem, which puts a major burden on health services and limits access to treatment [1]. Obesity is strongly associated with a number of health conditions, including type 2 diabetes, heart disease, cancer, stroke, and depression [1–3]. More severe obesity increases health risks, making access to obesity treatment essential [4, 5]. Group-based interventions, which can reduce the number of staff needed to deliver interventions, can improve accessibility to services and reduce weight times, and also provide opportunities for social support and sharing of strategies [3, 6]. Delivering group-based interventions digitally could improve their accessibility by reducing barriers for patient attendance and healthcare delivery. Previous research has examined online [7–11] and in-person group-based weight management interventions [12–14] separately, but there is a lack of synthesis of evidence of the impact of integrating the two strategies. This review aims to address this gap by providing a comprehensive review of the existing literature on online, group-based interventions for people with severe obesity.

The prevalence of obesity is increasing, particularly in the American and European regions, with 60% of European citizens reported to have obesity or overweight in 2022 [15]. In the United Kingdom, over 25% of adults have clinical obesity [4, 5]. Over a third of people with obesity report never having accessed any weight-management services and access to Tier 3 and 4 services - targeted for people with BMIs over 35 kg/m^2^ - can be even more difficult [16]. Lack of availability of services is an issue, with over 40% of Clinical Commissioning Groups not commissioning Tier 3 services, resulting in long waiting times and a lack of local resources for people who need them [16, 17]. For instance, in one study, nearly 75% of people with BMIs greater than 35 kg/m^2^ (and almost 60% of people with BMIs over 40 kg/m^2^) did not access any obesity services over a 7 year period [18]. Obesity is strongly associated with a number of noncommunicable health conditions (such as type 2 diabetes, cardiovascular conditions, and cancer [1, 19, 20]), with 40% of people with obesity having high or very high health risks, with increased risk in women [5]. Behaviours associated with obesity, such as an unhealthy diet and reduced physical activity, are commonly known to link obesity with these conditions [21]. Behavioural weight management interventions can help address some of the contributing factors for these conditions, and accessing them in a timely manner is important to reduce the risk of long-term complications [21, 22].

Delivering behavioural interventions in a group-based setting can help improve availability of services by reducing the number of staff needed to deliver interventions. When delivered face-to-face, group-based interventions have generally been found to be effective at supporting weight management [12, 13], potentially even superior to individual interventions [23–25]. The challenge with group-based interventions is that they can be difficult to offer and access in-person due to time, budget, travel, or facility constraints. Since the Covid-19 pandemic, the use of digital technology is becoming increasingly common in healthcare [26]. Evaluations of digital interventions for weight management have yielded mixed results [7–11]; some research suggests that digital interventions are more effective than no intervention, but less so than face-to-face interventions [27].

Integrating digital and group-based approaches to weight management interventions has the potential to further improve access by reducing burden on services and wait times for patients. Despite this, no published or planned systematic reviews (registered in PROSPERO) synthesising evidence around online, group-based interventions were identified (detailed in our published protocol [28]). This demonstrates a gap in the literature and the need for a comprehensive overview of online, group-based interventions for people with severe obesity. If group-based interventions can be delivered to the same or better effect online, this could enable more patients to access support in a more timely manner. Examining how these interventions are being evaluated and what evidence there is of their effectiveness at achieving health and behavioural outcomes will inform future intervention development and evaluation.

## Aims and research questions

The primary aim of this review was to synthesise the available evidence about the effectiveness and user perceptions of online, group-based interventions for adults with severe obesity. Based on the previous literature around group-based and digital interventions, we hypothesised that online, group-based interventions would have the potential to be effective at supporting weight management but that there may be barriers to sustained engagement with them. To achieve this aim, the review was centred on two research questions:

1. What methods of delivering online, group-based behaviour change interventions for adults with severe obesity are the most effective at establishing and maintaining positive health behaviour changes and weight loss?
2. What are the perceptions of the acceptability, usability, and overall user experience for different online, group-based behaviour change interventions for adults with severe obesity?

## Methods

### Overview

The population, intervention, comparator, outcome, study type (PICOS) framework was used to structure the scope of the review [29] and the report adheres to the Preferred Reporting Items for Systematic Reviews and Meta-Analyses (PRISMA) guidelines [30]. The PRISMA checklist is included in Supplementary Table S1. The methods are detailed in a previously published protocol [28].

### Eligibility criteria

The PICOS framework (Table 1) was defined based on the research questions and used to structure the scope of the study and the eligibility criteria.

**Table 1:** PICOS framework.

Population	Adults (≥18 years) with severe obesity (BMI ≥ 35 kg/m^2^)
Intervention	Online, group-based interventions aiming to change obesity-related behaviours (physical activity and dietary behaviour)
Comparator	The review included studies with and without a comparator; there were no restrictions on the type of comparator, if studies included one (e.g. face-to-face, phone, or other online group-based or individual interventions, waitlist control, etc.)
Outcomes	The primary outcome was the interventions' effectiveness at supporting behaviour change and weight loss. Secondary outcomes included engagement, patient experience, and study and intervention characteristics.
Study types	To avoid excluding relevant studies and to capture the variety of outcomes we wanted to examine, any type of study evaluating an online, group-based intervention for people with severe obesity was eligible (e.g. randomised controlled trials, quantitative, qualitative, cohort, and case studies). Reviews, protocols, and papers that described interventions without evaluating them were excluded.

### Search strategy

The search was executed in five databases: MEDLINE, Embase, CINAHL, Web of Science, and the Cochrane Central Register of Controlled Trials (CENTRAL). We chose not to search grey literature to ensure that the studies included had been peer-reviewed and should be of reasonable quality. In a change to the protocol, the databases ‘APA PsycINFO’ and ‘ProQuest Dissertations and Theses’ were not searched due to website unavailability. An initial review of the literature was used to identify key terms and develop the search strategy (detailed in the published protocol [28]). The search string was based on three key themes joined with the following structure: online (MeSH OR Keywords) AND group-based (MeSH OR Keywords) AND severe obesity (MeSH OR Keywords).

The original search was conducted in March 2021, but the screening and analysis of the results was delayed. To allow for this delay, the searches were re-run in April 2022 and again in May 2024. See Supplementary Table S2 for a record of all of the searches and results at all three time points.

### Inclusion criteria

Studies that examined online, group-based interventions for adults (18 years or older) with severe obesity were eligible for inclusion; however, to ensure inclusion of all relevant studies, those with participants below 18 years of age were included as long as the means and standard deviations of ages of the study samples indicated that a majority of participants were adults (Box 1). The scope of this review was focused on people with severe obesity because of the evidence of their greater inaccessibility to services [16–18]. Severe obesity was defined as BMI ≥ 35 kg/m^2^; however, to ensure that relevant studies were not excluded, study populations that included a range of BMIs beyond the ≥35 kg/m^2^ limit were also included, if the mean BMI at the start of the study was greater than or equal to 35 kg/m^2^. “Group-based” interventions were defined as interventions that were primarily group-based (involving 3 or more participants), although group interventions with some independent elements were eligible for inclusion. Interventions were considered to be ‘online’ if they were enabled via technology to connect participants, whether or not they were ‘live’ (i.e. synchronous or asynchronous).

A broad range of studies were eligible for inclusion, including randomised controlled trials, quantitative, qualitative, cohort, and case studies as long as they evaluated the intervention, with or without a comparator, and reported outcomes. Outcomes could include behaviours (such as eating, physical activity, etc.) or anthropometric measures. Studies published in any year were eligible for inclusion.

### Exclusion criteria

Any studies that did not evaluate the intervention (e.g. protocols, posters, conference abstracts, reports, or intervention descriptions) were excluded; reviews were also excluded due to time and resource restrictions. Studies examining child, parental, or family interventions that focused primarily on childhood obesity outcomes were also excluded, as were dietary or physical activity interventions that had a primary purpose other than managing obesity (e.g. supporting rehabilitation, chronic obstructive pulmonary disease, diabetes, etc.). Studies where the mean starting BMI of the sample was <35 kg/m^2^ were also not eligible. Papers for which the full-texts were not accessible were also excluded from this review.

### Screening and article selection

All references were retrieved and stored in EndNote X9 for duplicate removal and then uploaded into the Rayyan systematic review software. Title and abstract screening and full-text screening was conducted independently by two authors against the eligibility criteria, with disagreements resolved by consensus. The updated search and screening in 2024 was conducted by one author. The screening and selection process details were recorded in a PRISMA flow diagram.

### Data extraction

Two independent reviewers extracted data from included studies based on the predetermined data extraction form (Box 1) [28]. Any disagreements were discussed and resolved by consensus. The 2024 update was conducted by one reviewer.

Box 1 Data extraction items

**Table:** 

**General study information**
• Year of publication
• Country of study
• Sample demographics (mean BMI, mean age, gender proportion)
• Initial sample size (total)
• Initial sample size (online intervention group)
• Analysed sample size (online intervention group)
**Intervention**
• Online platform
• Aim of study
• Group size
• Number of intervention sessions
• Length of intervention sessions
• Intervention duration
• Follow-up time points
• Theory the intervention was based on (if any)
**Evaluation**
• Outcomes measured
• Effect of intervention on behaviour change outcomes
• Effect of intervention on health outcomes (eg, weight, BMI)
• Participant engagement (eg, drop-out rates, number of sessions attended)
• Acceptability
• Usability
• Participant satisfaction/feedback
• Other key performance indicators reported
• Facilitators to group engagement
• Barriers to group engagement

### Quality appraisal and risk of bias assessment

Risk of bias was evaluated using the Cochrane Collaboration Risk of Bias 2 (RoB 2) tool for randomised controlled trials [31, 32]. In the protocol [28], we planned to use the Risk Of Bias In Non-randomised Studies - of Interventions (ROBINS-I) tool [33] for non-randomised trials, but given the wide range of study methodologies included, the Mixed-Methods Appraisal Tool (MMAT) was used instead [34]. Quality appraisal and RoB assessments were conducted by three authors.

### Data analysis and synthesis

Study and intervention information was summarised in tables. Descriptive analyses using counts and percentages were used to quantitatively synthesise intervention outcomes. Narrative analysis was used to synthesise qualitative data relating to acceptability, usability, patient feedback, and factors influencing engagement. A meta-analysis was conducted on eligible studies to assess the impact of online, group-based interventions on the primary outcome, weight change (in kg). The effect was estimated based on change scores (from baseline to post-intervention). For studies that reported weight at baseline and at post-intervention rather than weight change over the intervention period, mean weight change was calculated using subtraction and the standard deviation (SD) was calculated using the formula: SD_change_ = (SD^2^_baseline_ + SD^2^_final_ − 2*Corr*SD_baseline_*SD_final_)^^0.5^ [35], assuming a conservative value of 0.5 for the correlation coefficient. A random-effects model was used as heterogeneity was present and statistical heterogeneity was measured via a Tau-squared test and the *I*^*2*^ measure. Publication bias was assessed using the Egger’s test [36]. Results were presented as a forest plot for weight change. The meta-analysis was conducted using the IBM SPSS Statistics software.

## Results

### Included studies

**Fig. 1:**
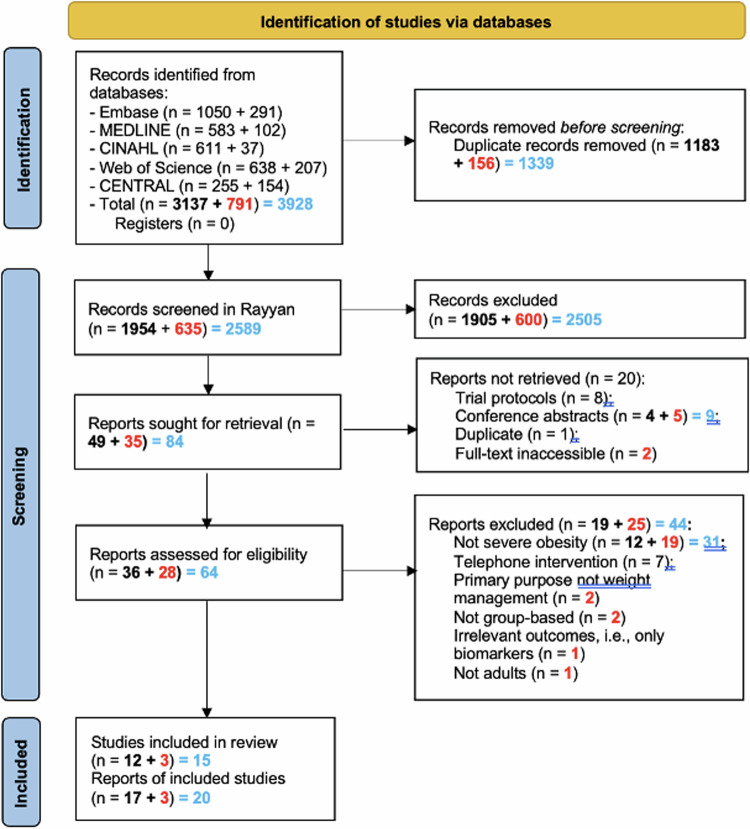
PRISMA flow diagram.

From the initial two search rounds (March 2021 and April 2022), 3137 articles were retrieved from five databases. 1183 duplicates were removed before screening using the EndNote X9 duplicate removal feature. After title and abstract screening, 49 articles remained. At this stage, full reports of trial registrations and conference abstracts were sought. Six mapped to an identified reference within the search results. Five were not associated with a full published report. One was an ongoing trial, and one was a duplicate. In total, 13 reports were not retrieved (Fig. 1) and 36 full texts were screened, resulting in 17 included papers that described 12 different studies. Four papers reported on one study [37–40] and three papers on another [41–43]. The data was separately extracted from each paper because many of the papers had different numbers of participants (e.g. subgroup analyses).

The updated search in May 2024 identified 952 articles published since the previous search (2022–2024). After duplicate removal, the titles and abstracts of 635 references were screened. Of these, 35 were selected for full-text review, 3 of which were included, for a total of 20 papers describing 15 different studies.

### Study characteristics

The study characteristics of the 20 included reports are summarised in Table 2. Studies were conducted between 2005 and 2023 with total sample sizes ranging from 14 to 481. As many of the studies were multi-arm trials, we have reported the initial and analysed sample sizes for the online, group-based intervention in addition to the total sample. Analysed sample sizes for that condition specifically ranged from 11 to 418. Almost all of the studies had sample populations that were mostly female (the median was 90% female for the 17 papers or 82% for the 15 studies) and all but 3 studies had more female participants than male (one of the studies had only male participants as the target population was men [44]). Mean BMIs at baseline were measured in clinic or using scales provided to study participants and ranged from 35.2 kg/m^2^ to 49.2 kg/m^2^.

**Table 2:** Summary of study and baseline sample characteristics.

Citation	Study year	Study design	Country	Total sample	Online int. group initial sample	Online int. group analysed sample	Mean BMI (kg/m^2^)	Mean age	Female (%)
Abbott et al. [57]	2020	Prospective cross-sectional study	England (UK)	315	227	227	46.8	44	70
Ahrendt et al. [45]	2010	Retrospective cohort study	USA	120	60	60	38.9	57	8
Batsis et al. 2021 [51]	2021	Non-randomised, non-blinded, single-arm study	USA	53	53	44	36.5	72.9	82
Bernhart et al. [46]	2020–2021	Feasibility study	USA	60	Synchronous: 43; Asynchronous: 17	–	36.8	48.3	Synchronous: 78; Asynchronous: 100
Bruce et al. [47]	2020–2022	Randomised Controlled Trial	USA	71	36	34	37.5	47	82
Cavallo et al. [52]	2012	Single group pre-post design feasibility study	USA	40	40	12	39	30	100
Cliffe et al. [53]	2020	Qualitative	Wales (UK)	14	13	13	39.3	48.5	61
Fraticelli et al. [48]	2020	Randomised Controlled Trial	Italy	36	18	11	35.2	56.5	50
Griffith et al. [44]	2019–2020	Pilot RCT	USA	72	72	58	37	50	0
Harvey-Berino et al. [37]	2005	Randomised Controlled Trial	USA	481	320	303	35.6	46.7	93
Harvey-Berino et al. [38]	2005	Randomised Controlled Trial^a^	USA	323	323	234	35.6	46.7	93
Krukowski et al. [40]	2005	Randomised Controlled Trial	USA	323	162	161	35.7	46.3	92
Krukowski et al. [39]	2005	Randomised Controlled Trial^b^	USA	161^b^	161	161	35.6	46.2	92
Stansbury et al. [41]	2016–2018	Secondary analysis of the control arm in a randomised trial	USA	212^c^	212	194	35.8	48	91
West et al. [42]	2016–2018	Randomised Controlled Trial	USA	418	418	360	35.7	49	91
West et al. [43]	2016–2018	Randomised Controlled Trial	USA	418	418	418	35.7	49	91
West et al. [54]	2010–2014	Randomised Controlled Trial	USA	73	37	35	35.8	50.5	92
West et al. [55]	2020	Pilot RCT	USA	398	398	323	36	48.4	90
Wild et al. [49]	2012	Randomised Controlled Trial	Germany	117	59	58	49.2	41.2	60
Willis et al. [50]	2016	Pilot RCT	USA	70	34	–	36.8	46.8	85

^a^Part of the Harvey-Berino 2010 study (23).

^b^Same study as the Krukowski 2011 study [40]. This paper focuses on the 161 participants who were randomised to the online condition.

^c^This subgroup analysis is one arm of the West 2020 study [42].

Of the various outcome measures reported, weight was the most common, reported in all but 5 of the 20 papers and all but 3 of the 15 studies. Eight studies used other weight-related measures, including BMI, waist to hip ratio, waist circumference, and fat percentage [40, 44–50]. Other outcomes measured in the studies included quality of life and mental health-related outcomes (including depression, eating disorder, and late life function) [45–47, 49, 51], participant experience regarding acceptability and barriers [44, 45, 51–53], self-efficacy [45, 49], and engagement (including attendance, self-monitoring, and completion) [39, 43, 44, 51, 54]. Several studies also measured key obesity-related behaviours such as physical activity (which also included changes in ability, such as the 30 s sit to stand test, mobility, and walk and grip strength) [41–43, 47, 51, 52] and dietary behaviours (such as adherence to a mediterranean diet (PREDIMED scores), energy and macronutrient intake, diet quality, and diet self-efficacy) [42, 43, 46, 48, 50, 52]. Two studies also collected biometric data, specifically blood pressure [44, 48], total cholesterol [44], capillary fasting blood glucose, and venous HbA1c [48], and another study examined the accuracy of participants’ self-reported weight measurements [38]. The full data extraction table is shown in supplementary material (Supplementary Table S3).

### Intervention characteristics

The characteristics of the interventions are summarised in Table 3. There was considerable heterogeneity in the reported characteristics of these studies. There was a range in group sizes, from 3 to 43 participants, although most of the studies had group sizes of less than 20 (9/15, with 4 studies not reporting group size). The number of sessions provided also ranged widely, from 6 to 47. Only 60% of the papers (12/20) reported session length, and these ranged from 45–75 min. There was more similarity in the duration of the intervention, with approximately half of them lasting for 6 months (8/15 studies). Of the remaining studies, 5 lasted for 3–4 months, 1 lasted for 12 months, and 2 lasted for 18 months.

**Table 3:** Summary of intervention characteristics.

Citation	Platform	Group size	# of sessions	Length of sessions	Duration of intervention
Abbott et al. [57]	Vidyo Connect	*NR*^*a*^	6	1 h	6 months
Ahrendt et al. [45]	Video-conference	8 ×3 = 24	12	1 h	12 weeks
Batsis et al. [51]	HIPAA compliant version of Zoom	10	47	1 h	6 months
Bernhart et al. [46]	Zoom	17/43	12	75 min	3 months
Bruce et al. [47]	Zoom	10–15	24	1 h	6 months
Cavallo et al. [52]	Facebook	40	8	*NR*	16 weeks
Cliffe et al. [53]	Skype for Business	6	10	*NR*	6 months
Fraticelli et al. [48]	Web-based ‘classroom’ platform	up to 10	3	*NR*	6 months
Griffith et al. [44]	GoToMeeting or Zoom	NR	12	45 min	3 months
Harvey-Berino et al. [37]	Online via a synchronous chat group	15–20 (says 12–18 elsewhere)	24	1 h	6 months
Harvey-Berino et al. [38]	Online via a synchronous chat group	15–20 (says 12–18 elsewhere)	24	1 h	6 months
Krukowski et al. [40]	Website	12–18	24	1 h	6 months
Krukowski et al. [39]	Website	12–18	24	1 h	6 months
Stansbury et al. [41]	*NR*	*NR*	24	*NR*	6 months
West et al. [42]	Zoom	17–20	16	1 h	4 months
West et al. [43]	*NR*	*NR*	36 or 42	*NR*	18 months
West et al. [54]	*NR*	*NR*	24	*NR*	6 months
West et al. [55]	*NR*	*NR*	24	*NR*	18 months
Wild et al. [49]	Videoconference	3	6	50 min	1 year
Willis et al. [50]	Facebook	12–18	24	*NR*	6 months

^a^*NR* not reported.

Less than half of the studies (7/15) reported that their interventions were based on any specific theory. Five of the interventions used the Social Cognitive Theory [46, 47, 50, 51, 55]. Other theories used included the Self-Determination Theory [44, 53], the technology acceptance model [51], problem-solving and relapse prevention models [50], and self-regulation approaches [55].

### Aim 1: Impact and effectiveness of interventions

The outcome measures and key findings of each of the included studies are summarised in Table 4. Common outcome measures included weight-related outcomes (e.g. weight loss, BMI, waist-to-height ratio), participant perceptions of the intervention, engagement, and behavioural outcomes (e.g. physical activity, dietary habits).

**Table 4:** Summary of study outcomes.

Citation	Study aims	Outcome measures	Key findings
Abbott et al. [57]	To assess the uptake of transfer from a face-to-face to a virtual group weight management programme during COVID-19 pandemic and investigate predictors of uptake	Acceptance of virtual group	• Reasons for lack of acceptance: lack of internet access (89.8%), preference for face-to-face sessions (10.2%) • Older and BAME patients less likely to engage
Ahrendt et al. [45]	To determine the effectiveness of delivering the MOVE! Weight Management Program using videoconferencing technology	Weight-related: Weight, BMI Wellbeing: QoL, Depression, eating disorder Behavioural: Self efficacy	Intervention group lost weight while control gained weight (mean difference in weight loss between groups: 5.5 kg)
Batsis et al. [51]	To assess the feasibility, acceptability and preliminary outcomes of an integrated technology-based health promotion intervention in rural-living, older adults	Feasibility & acceptability Completion & attendance Changes in weight & movement (30STS), walk & grip strength, disability	• High satisfaction with the trial • Mean weight loss: 4.6 ± 3.5 kg (4.7%) - 50% of cohort had clinically significant improvement • 30STS improved from 13.5 ± 5.7 to 16.7 ± 5.9 repetitions; clinically significant changes in 6-min walk: 42.0 ± 77.3 m; Improvements in total, upper, basic lower, and advanced lower extremity function • No change in gait speed and grip strength
Bernhart et al. [46]	To assess the reach and effectiveness of a virtual vegan diet intervention for African Americans	Weight-related: Weight, BMI Diet-related: Diet self-efficacy, Diet quality Well-being: QoL	• Synchronous and asynchronous groups had significant reduction in weight and BMI • QoL increased significantly in asynchronous group only • Self-efficacy significantly increased in both groups; self-perception of health increased in asynchronous only
Bruce et al. [47]	To examine the efficacy of a behavioural weight loss intervention for people with Multiple Sclerosis and obesity	Weight-related: Weight loss, BMI, waist-to-height ratio, % fat tissue Behavioural: Physical activity, mobility, Well-being: fatigue, QoL	• Statistically significant difference between groups in activity (increased in IG, decreased in TAU group); no statistically significant difference in mobility • Statistically significant difference in weight loss (higher in IG vs TAU), BMI and waist-to-height ratio • Mental QoL increased in IG vs TAU but not physical QoL
Cavallo et al. [52]	To examine the feasibility of delivering a group-based weight-loss intervention adapted to low-income women of reproductive age using Web-based educational content and social media.	Weight-related: Weight, Behavioural: Physical activity, dietary habits Intervention-related: acceptability, barriers to use	• Mean change in walking time: 116.3 minutes/week (SD = 191.6 minutes/week); mean change in servings/day of fruit and vegetables was 0.5 servings/day (SD = 1.5 servings/day) • Mean weight change in completers was -1.3 kg (SD 4.4 kg); 7/12 participants lost weight. • Lack of access to internet, intervention components on multiple platforms, lack of prior familiarity with other participants were reported as issues • Feedback: Additional tools, more frequent group sessions, more reminders, and tips to improve group cohesion
Cliffe et al. [53]	To understand participant experience of accessing an adapted programme via videoconference	Qualitative experience	• Seven behaviour change themes: Personal responsibility; decision ownership; connectedness; identifying; peer support; new strategies; favourable comparisons.
Fraticelli et al. [48]	To compare a web-based nutritional intervention versus a traditional one, before and during the Italian ‘lockdown’ period, in overweight and obese participants affected by T2D or impaired glucose regulation (IGR)	Weight-related: BMI data, Waist-to-height ratio Blood markers: capillary fasting blood glucose, venous HbA1c, blood pressure and PREDIMED scores	• Progressive weight loss and improvement of BMI; decrease in median WHR • No significant differences for other outcomes
Griffith et al. [44]	To assess the feasibility, acceptability, and impact of a behavioural virtual weight loss intervention tailored for middle-aged African American men	Feasibility, acceptability Weight-related: Anthropometric measures Blood markers	• No significant within group changes for eating practices or physical activity. Fruit and vegetable consumption and vegetable intake increased for intervention group. • Small but significant decrease in BMI. Weight and body fat decreased but not significant • Only some participants felt supported by other group members
Harvey-Berino et al. [37]	To evaluate the efficacy of an Internet behavioural weight loss program; and determine if adding periodic in-person sessions to an Internet intervention improves outcomes	Weight loss	• Reductions in calorie intake in all groups (inc. f2f) • In person weight loss significantly greater than internet and hybrid groups; proportion losing 7% did not differ significantly between groups • In person participants perceived group support to be significantly higher than internet but hybrid did not differ from either; working alliance raated similarly
Harvey-Berino et al. [38]	To understand the accuracy of self-reported weight over a 6-month Web-based obesity program	Accuracy of self-reported weight measurements	• Significant difference between weight loss calculated using reported weight vs observed weight - reported weight change larger than observed. This also differed significantly by race (higher reported in African American vs white) and by condition (lower for internet vs hybrid) but not gender.
Krukowski et al. [40]	To assess the costs associated with a group behavioural weight loss intervention and compare cost-effectiveness based on treatment delivery modality (in-person vs. Internet).	Weight, BMI	• Participants in the Internet condition lost an average of 5.5 ± 5.6 kg
Krukowski et al. [39]	To examine patterns of self-monitoring associated with greater weight loss at 6-months.	Weight, self-monitoring	• Mean weight loss of 5.5 ± 5.6 kg at six months • Female participants significantly less likely to log in to the self-monitoring tool than male; greater self-monitoring for older participants • Overall logins were significant predictor of weight loss
Stansbury et al. [41]	To characterise individuals with distinct patterns of weekly adherence to Physical Activity goals.	Adherence to physical activity goals	• Greatest weight losses in subgroup likely to meet program goals for weekly minutes of moderate-to-vigorous PA and daily steps.
West et al. [42]	To assess whether adding financial incentives for self-monitoring and achieving target weight losses increases weight losses attained in a fully online, group-based behavioural weight management program compared with the same program alone	Weight loss Engagement (attendance, self-monitoring of body weight, dietary intake, and physical activity)	• Incentives group lost more weight than no incentives • Treatment engagement and study retention higher in incentives condition
West et al. [43]	To examine the impact of an integrated incentive scheme and to explore what happens to weight outcomes and critical weight management behaviours once financial incentives end.	Weight loss Engagement (attendance, self-monitoring of body weight, dietary intake, and physical activity)	• Greater weight loss associated with financial incentives at 6 months, with significantly greater proportion achieving clinically meaningful weight loss (but not statistically significant). • This result no longer apparen at month 12 but significantly greater proportion of incentives group were weight stable from month 6 to month 12
West et al. [54]	To examine whether the addition of online motivational interviewing (MI) chats to a web-based, group behavioural obesity treatment program augments weight loss outcomes relative to the web-based weight control program alone.	Weight loss Attendance, self-monitoring	• No significant differences in weight loss between groups with and without motivational interviewing • No difference in self-monitoring or attendance between groups
West et al. [55]	To assess value of feedback type incorporated into online groups	Weight loss	• Those receiving pre-scripted feedback lost more weight than those receiving tailored feedback.
Wild et al. [49]	To assess the efficacy of interventions after bariatric surgery. The online intervention was aimed at training strategies, and skills to improve coping, stress management and relaxation.	Weight-related: Weight, BMI Wellbeing: QoL, Depression, eating disorder Behavioural: Self efficacy	• No differences between intervention and control in QoL, self efficacy, eating psychopathology and depressive symptoms. • Patients significantly reduced weight but no difference between the groups. • In subgroup of patients with depression, intervention significantly improved depression and QoL scores.
Willis et al. [50]	To evaluate the practicality and efficacy of using an online social network to deliver a weight management programme	Weight-related: BMI, Weight, Waist circumference Diet: energy and macronutrient intake	• Weight change (%) from baseline to 6 months was -5.8 (SD 6.7%) in online group • Participants in online group reported a lack of familiarization with other members limited their comfort of sharing information • Total cost per participant was lower in online vs telephone group

#### Health outcomes

Less than half of the studies (5/15) reported significant differences between conditions in terms of weight loss metrics. Three of these directly compared the effect of the study intervention against a comparator and found different results. One, a retrospective cohort study, reported that the group receiving a video conferencing-based intervention lost weight while the control group, who did not receive any intervention, gained weight [45]; the second, an RCT with low risk of bias, found that in-person intervention was associated with significantly greater weight loss than internet and hybrid interventions [37]. The third study, another RCT with low risk of bias, found that a telehealth intervention resulted in statistically significant weight loss compared to treatment as usual [47]. The other two studies examined the difference between specific features of online, group-based interventions. One RCT with low risk of bias found that participants in a condition with incentives lost significantly more weight (and achieved clinically meaningful loss ≥5%) than those without. The difference in total weight loss between groups was no longer significant at 12 months, despite ongoing incentives, although significantly more of the incentive group participants had stable weights at 12 months [42, 43]. The other study, a pilot RCT with low risk of bias, found that participants in the version of the intervention with pre-scripted feedback (compared to counsellor-crafted feedback) lost significantly more weight [55].

A fifth of the studies (3/15) reported finding no significant difference in weight loss between conditions; in two cases, the online intervention was compared with conventional delivery of the service [49, 53] and in the other, the addition of motivational interviewing did not improve outcomes compared to the standard web-based program [54]. The remaining studies (7/15) reported weight loss over time but did not compare weight loss in the online, group-based intervention with another condition [39, 40, 44, 46, 48, 50–52].

#### Behaviour change outcomes

Seven studies reported on physical activity or dietary behaviour change as well as health outcomes. One pilot study found a mean change in weekly walking time of 116 min, although there was a huge range and standard deviation in their small sample of 12 participants [52]. Another found significant improvements in the 30 second sit-to-stand and 6 minute walk tests, but not in gait speed or grip strength [51] and a third found that there were higher rates of positive behaviours when the online intervention included incentives compared to when it did not [42]. An RCT found a significant difference between the intervention and treatment-as-usual group in accelerometer data (activity increased for the intervention group and decreased in the control group) but no significant difference between groups in mobility [47], while another, a pilot RCT, found no significant within-group changes for physical activity [44].

Three studies reported on dietary outcomes, of which two did not find a difference between groups (on calorie intake, which was reduced in all conditions [37], or on eating psychopathology [49]). The third study found no significant within group changes in eating practices, although fruit and vegetable intake did increase in the intervention group.

#### Meta-analysis of impact on mean weight change

**Fig. 2:**
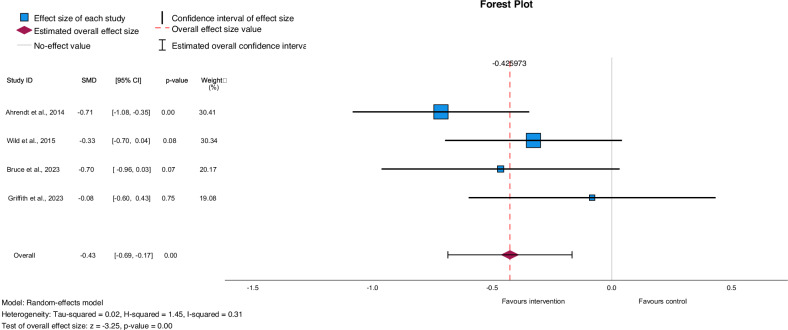
Forest plot for mean weight change in intervention group compared to waitlist/standard care.

Of the 15 identified studies, 13 reported weight change and 9 of these were RCTs; however, only four [44, 45, 47, 49] had consistent comparison groups (waitlist or standard care) and were sufficiently similar to conduct a meta-analysis. The remaining studies had various comparisons - including between online and in-person group-based interventions [40, 48], the intervention delivered with and without additional components [42, 54, 55], and a social-media based intervention compared to a conference call [50] - but they were determined to be too heterogeneous for a meta-analysis to be meaningful and too few to conduct separate meta-analyses [56]. The studies included in the meta-analysis compared the intervention with either a waitlist control or standard care and included a total of 356 participants (179 intervention, 177 control). Three of the four included studies had conducted sample size calculations and were sufficiently powered to detect a reduction in weight of 3–10 kg with 80% power [45, 47, 49]. From this small sample, the results of the meta-analysis indicated that online, group-based interventions have a statistically significant effect on weight loss compared to no intervention or standard care with a small-to-moderate effect size (SMD (Cohen’s d) = −0.428; *p* = 0.001; 95% CI: −0.69 to −0.17) (Fig. 2); however, this should be interpreted with caution. Heterogeneity was small (*I*^2^ = 31%) and the Eggers’ test indicated no publication bias (*p* = 0.28).

### Aim 2: User experience with the interventions

#### Engagement

All but one of the papers [40] reported data relating to engagement. Most of the reported outcomes were related to completion rates, which ranged from 30% [52] to 95% [55] (data for each study is provided in Supplementary Table S3). Of the papers that reported comparisons of engagement between online and other conditions (e.g. traditional route, in person or hybrid), most found no significant difference [37, 48, 54]. One study did find significantly higher engagement with an internet-based intervention when incentives were provided (91% compared to 81% retention) [42]. Three studies examined how user characteristics were associated with engagement: one found that older and Black, Asian and Minority Ethnicity (BAME) patients were less likely to choose the online-based intervention when it was offered to them [57], while another found that older and male participants were more likely to engage in self-monitoring [39]. One study found that the most common reasons for declining to participate in the online intervention were a lack of digital skills or access to the internet [57].

All but three of the studies reported some information about facilitators to engagement in the group intervention. Approximately half of them (7/15) tailored the intervention in some way to improve engagement. Two studies [37, 51] added in-person group sessions alongside online sessions, but one of them [37] found no significant effect of this. The other [51] provided additional technical support, which was reported to have a positive impact on engagement. About a quarter of the studies (4/15) included interactive forums or group chat functions to improve engagement. Most studies also provided detailed technical instructions and run-throughs prior to the sessions, which minimised disruptions caused by technical issues. One study [53] established ground rules and reported this to have increased participants’ comfort and facilitated engagement. Another [42] provided financial incentives, which increased engagement. Two studies [52, 53] asked participants for engagement-related feedback and received suggestions to help improve engagement, including reminders of sessions, team building activities, and having someone familiar in the chat room.

Two-thirds of the studies (10/15) also reported barriers to group engagement. Common barriers included a ‘digital divide’ in internet accessibility (access to internet and usability of technology) and lack of familiarity with other group members. Two studies [52, 55] reported hectic schedules resulting in a lack of consensus for session times as a significant barrier to engagement. One study [52] had its components on multiple platforms which participants highlighted as a burden, while another [37] had a different counsellor in person versus online, which reportedly reduced the alliance. Another study [54] used text-based communication rather than video, which hindered engagement as the lack of verbal cues and absence of tone led to the text being easily misconstrued. In another study, the virtual environment itself was considered a barrier to social support and consequently, engagement, by some of the participants [44].

#### Acceptability, usability, and satisfaction

Six of the studies examined some outcome related to acceptability, usability, or satisfaction. Four studies reported positive outcomes related to patient experience: one intervention had high satisfaction ratings [51], one identified themes such as reduced travel, time, and cost burdens and a less stressful or daunting environment for introverts [53], another hypothesised that the use of an online intervention enabled continued engagement and connection throughout the Covid-19 lockdown, mitigating its negative impact on health behaviours [48], and the fourth reported that receiving the intervention online was convenient [44].

Some studies also identified concerns with the online interventions. Two studies reported that a lack of familiarity limited participants’ comfort sharing information [50] and potentially also their engagement [52]. A couple of studies also identified technical issues such as difficulty accessing the Internet [52, 53] and managing intervention components on different platforms [52].

One study also collected participants’ suggestions for improvement, which included: digital calorie counting applications, food scales, more frequent sessions, more reminders and incentives, adding team-building exercises, inviting friends, and facilitating face-to-face interaction by connecting participants directly [52].

### Risk of bias and quality assessment

The risk of bias of the randomised trials was evaluated using the Cochrane Collaboration RoB 2 tool (Table 5) [31, 32]. Five of the nine studies were assessed as having low risk of bias in all domains and only one was considered to have high risk of bias. Of the various domains, the randomisation process had the most concerns, with three studies failing to report sufficient information about allocation, schedule generation, or concealment. Three studies also provided only limited drop-out information, although this was not thought likely to be related to the outcomes. Only one domain - measurement of the outcome - was assessed as high risk; this was because the patients measured their own BMI, which could have been affected by their knowledge of the intervention conditions. This was not the planned procedure for outcome measurement protocol; self-measurement of weight and circumference data was necessitated by Covid-19 lockdowns and participants had previously been instructed on how to take measurements themselves.

**Table 5:** Risk of Bias 2 assessment of randomised studies.

Study	Random-ization process	Deviations from intended interventions (assignment)	Missing outcome data	Measurement of the outcome	Selection of the reported result	Overall Bias
Bruce et al. [47]						
Fraticelli et al. [48]						
Griffith et al. [44]						
Harvey et al. [37], Harvey-Berino et al. [38], Krukowski et al. [40], Krukowski et al. [39]^a^						
West et al. [54]						
West et al. [42], West et al. [43], Stansbury et al. [41]^b^						
West et al. [55]						
Wild et al. [49]						
Willis et al. [50]						

refers to ‘low risk of bias’,  refers to ‘some concerns’,  refers to ‘high risk of bias’

^a^These four references refer to the same RCT.

^b^These three references refer to the same RCT.

The Mixed-Methods Appraisal Tool (MMAT) [34] was used to assess the quality of the remaining five studies. Generally, the studies met the criteria, with the exception of confounders being accounted for in the design and analysis of the study. The full assessment (with comments) for all of the 12 studies is provided in Supplementary Table S3.

## Discussion

### Principal findings

Fifteen studies were identified that examined the impact of online, group-based weight management interventions for people with severe obesity (BMI ≥ 35 kg/m^2^). There was substantial variability among the interventions, in terms of online platform (including videoconferencing, websites, and chat functions), group size, and number of sessions, but they most commonly lasted for around 6 months and had 1 hour sessions. Overall, the evidence supported our hypothesis that online, group-based interventions could have a positive impact on weight management, as most of the studies observed weight loss associated with participating in the intervention. A meta-analysis also indicated a small-to-moderate effect of the interventions at reducing weight compared to no intervention. Drawing implications from these findings should be done cautiously, for a couple of reasons. Only four of the 15 studies were sufficiently comparable to be eligible for the meta-analysis and many of the studies had small and non-representative sample sizes (9/15 had samples of less than 100 participants and most of the studies had predominantly female participants). Additionally, although less than half of the studies (7/15) looked at behavioural outcomes, the evidence of impact of the interventions was limited, particularly for dietary behaviour. This is a common limitation in digital health research [58, 59], often due to barriers such as motivation, a perceived lack of social connection, and limitations with the recruitment methods [60].

With the available evidence, it was difficult to draw strong conclusions around the first research question of what methods of delivering online, group-based behaviour change interventions for adults with severe obesity are most effective. The relatively small set of studies included in this analysis made a subgroup analysis of intervention methods infeasible. For the remaining studies, the heterogeneity in the intervention types and the variety of comparisons conducted in the RCTs meant that there was little evidence to support any particular type of intervention. Some results suggested that adding incentives and using pre-scripted modular feedback can help support weight loss but that adding motivational interviewing might not increase the impact of the intervention. However, the small number of studies, heterogeneity, and flaws in design, all serve to limit the conclusions that can be drawn.

We were better able to address the second research question, which focused on users’ perceptions about different online, group-based weight management interventions, although our findings are limited by the fact that less than half of the studies (6/15) examined user experience. Online, group-based interventions were considered to be more convenient, but we identified several major barriers to engagement - including lack of access to fast internet, usability issues, and digital literacy issues - that prevented some people from participating and caused frustration in others.

### Implications for practice and future intervention design

To mitigate barriers related to usability of the specific platforms used, most studies provided detailed instructions and run-throughs prior to the sessions, both of which were considered helpful by participants. Organising times for the sessions that would be convenient for all participants was difficult in some studies, but having set dates and times for the sessions made attendance easier [52, 55]. A lack of familiarity with other group members had a negative impact on group cohesion, as did having different facilitators for different sessions [37, 50, 52].

The reviewed studies used a variety of methods to improve engagement, but only a few methods demonstrated positive impact in more than one study. One such method was sending the week’s content to participants ahead of the sessions, making them feel prepared and increasing comfort levels as participants knew what to expect. Attempts to remove technical barriers also largely had positive results, with participants giving positive feedback about early run-throughs. Incentives such as weekly tips and tricks, access to recipes, and detailed feedback from facilitators have also been reported as helpful. Feedback from participants included suggestions to provide reminders about the sessions and to include activities to promote participation and group cohesion [52, 53] (Table 6).

**Table 6:** Key strategies for improving user experience and engagement with online, group-based weight management interventions.

Strategy	Mechanism
Provide detailed instructions and/or run-throughs of the online platform	Helps address barriers relating to participants’ knowledge of and ability to use the online platform
Set dates and times for sessions	Ensures clarity around session times and enables planning of schedules
Maintain a consistent facilitator throughout sessions	Supports familiarity and group cohesion
Activities to build familiarity among group members	Supports group cohesion
Provide session content in advance	Establishes participants’ expectations, increasing comfort and reducing uncertainty
Provide additional content as incentives	Supports ongoing engagement in between sessions
Reminders about sessions	Helps support engagement by ensuring a session is not accidentally missed

### Comparison with existing literature and implications for future research

Several previous systematic reviews of web-based weight management interventions have found similar results. Generally, there appears to be some weak to moderate evidence of a positive effect of web-based interventions on weight loss, at least in the short term, although these analyses have been hampered by small sample sizes and variable methods [7, 61, 62]. Even though these reviews did not focus specifically on severe obesity or group-based interventions, they also only found small numbers of eligible studies - 8 [62], 9 [61], and 11 [7].

Evidence on the effectiveness of group-based weight management interventions (with various types of delivery) is stronger [12, 13]. Interestingly, one meta-analysis found that men-only groups had significantly higher weight loss than mixed-gender or women-only groups [12]. The review mentioned that this finding was consistent with previous literature about men benefiting from group interventions, but did not speculate on the possible reasons for this difference. The majority of participants in the studies included in this review were women. The literature suggests that this could potentially be a factor affecting the effectiveness of the interventions, although this could not be examined with the limited evidence available in this review. Future research should further explore the relationship between gender and mode of intervention delivery.

The findings of this review related to technical barriers to engagement, with a greater negative impact on marginalised communities such as older individuals or those from minority ethnic backgrounds [57], aligns with existing literature on digital inequalities [63] and should be considered while designing new digital interventions. One way to bridge this divide could be via referral to digital literacy programmes such as the NHS Digital Health Champions programme [64] before the start of the intervention [57].

Future interventions should continue using mitigation strategies highlighted in the reviewed studies, such as providing technical instructions and allowing time for run-throughs. To help reduce the issue of lack of familiarity, future studies could introduce a social communication component to encourage better group cohesion, while limiting all activities to as few platforms as possible to reduce burden on participants [52]. Any delays between expressing interest and beginning the intervention would also need to be kept at a minimum to avoid participants losing motivation. Co-production activities aimed at getting patient inputs specifically discussing challenges faced and ideas to improve their comfort levels and motivation could potentially lead to better engagement in future interventions.

Given the growing shift towards digital healthcare, especially since the start of the Covid-19 pandemic, it will be essential to understand the participant and intervention characteristics that are associated with the greatest benefit from online, group-based interventions to enable intervention designs to be optimised and tailored for specific groups. It would also support personalised medicine by helping healthcare providers and patients to choose the type of intervention most likely to benefit them - in-person or online, individual or group - while increasing the availability of services. To this end, it will also be important for future studies to explore patients’ perceptions of the intervention and the facilitators and barriers that they experience.

### Strengths and limitations of the review

Some of the strengths of the review are that the search was conducted using broad search terms in several databases to minimise the likelihood of missing a relevant study, and that the screening was conducted by two independent authors. The review included a range of studies using different methods, which provided different perspectives on evidence around the impact and experience of online, group-based weight management interventions. This enabled us to conduct qualitative and quantitative analyses and synthesise barriers and facilitators to group engagement, providing a more holistic interpretation of the body of literature on these types of interventions. This analysis also enabled us to outline key factors that can support the successful implementation of these interventions, which can inform the future development and delivery of online, group-based interventions for people with severe obesity.

One limitation was that due to time and resource constraints, two authors shared the work of data extraction and risk of bias and quality assessment. Another limitation is that due to the considerable heterogeneity of study design, comparisons, populations, and reported outcome measures, only four of the studies could be included in the meta-analysis, making it difficult to generalise the results of the analysis and provide meaningful knowledge [65]. Additionally, one of the studies included in the meta-analysis was underpowered [44]. This was included despite the limitations of including underpowered studies in meta-analyses [66] due to the small number of studies eligible for this analysis. This is a common issue in meta-analyses [66] and limits the strength of the conclusions that can be drawn from it. The low sample size of a majority of the studies included in this review also limits the other conclusions, and all the results found in this systematic review must be treated with caution. Given that underpowered studies and low sample sizes are a common limitation of digital health research [58, 60], future reviews should adjust for this issue while conducting statistical analyses to address this limitation. Finally, the heterogeneity of intervention characteristics, population characteristics, and comparison types also limit the ability to conclude who might benefit most from such interventions.

## Conclusion

The purpose of this systematic review was to synthesise evidence on the effectiveness of, and user experience with, online, group-based interventions for people with severe obesity. The evidence was mixed, but overall suggested some positive effect of the interventions on weight loss. The results from the meta-analysis tentatively suggest a small to moderate impact of the interventions compared to wait list controls, but this was based on a small sample and there was insufficient evidence to determine how digital group-based interventions compare with individual interventions (online or in-person) or face-to-face group-based interventions. Due to the heterogeneity of interventions and analysis types, it was difficult to conclude what specific intervention characteristics had the strongest impact on weight loss, but we identified key barriers to engagement including internet accessibility, digital literacy, a lack of time, and lack of familiarity with group members and the counsellor. Some of these barriers were mitigated by specific efforts made by study teams including detailed technical run-throughs and additional in-person sessions to improve group cohesion, but they were not always effective. Future studies into such interventions would benefit from co-production activities with diverse groups emphasising the inclusion of marginalised communities, specifically focusing on mitigating the identified barriers and further facilitating engagement. To improve access to and reduce the strain on weight management services, there is a need for further investigation in how to implement digital group-based interventions to maximise their potential benefits, which could inform decision and policy-making regarding obesity services.

## Supplementary information

Supllementary material

Table S3_Data extraction and risk of bias
