# Supplementary material for: The effectiveness and usability of online, group-based interventions for people with severe obesity: a systematic review and meta-analysis
Source: Int J Obes (Lond). 2024 Nov 19;49(4):564–77. doi: 10.1038/s41366-024-01669-2 (PMC11999869; doi:10.1038/s41366-024-01669-2)
Supplement: Supplementary file 1 — Supllementary material [file 41366_2024_1669_MOESM1_ESM.pdf]

The effectiveness and usability of online, group-based interventions for people with severe obesity: A systematic review and meta-analysis

Supplementary information

Madison Milne-Ives, BAS, MSc,[1,2] Lorna Burns, BA, MSc, MSc,[3] Dawn Swancutt, BSc, MPH, PhD,[4] Raff Calitri, BSc, MSc, PhD,[5] Ananya Ananthakrishnan, BTech, BA, MSc[1], Helene Davis[6], Jonathan Pinkney, BSc, MB BS, MRCP, MD, FRCP,[4] Mark Tarrant, BA, PhD,[7] Edward Meinert, MA, MSc, MBA, MPA, PhD, CEng FBCS EUR ING[1,2,8]

[1]Translational and Clinical Research Institute, Newcastle University, Newcastle upon Tyne, UK

[2]Centre for Health Technology, School of Nursing and Midwifery, University of Plymouth, Plymouth, United Kingdom, PL4 6DN

[3]Peninsula Dental School, Faculty of Health, University of Plymouth, Plymouth, United Kingdom, PL4 6DN

[4]Peninsula Medical School, Faculty of Health, University of Plymouth, Plymouth, United Kingdom, PL4 6DN

[5]Department of Health and Community Sciences , University of Exeter, Exeter, United Kingdom, EX1 2 LU

[6]Department of Clinical and Biomedical Sciences, University of Exeter Medical School, St Luke's Campus, Exeter, EX1 2LU

[7]School of Psychology, Faculty of Health, University of Plymouth, Portland Square, Plymouth, United Kingdom, PL4 8AA

[8]Department of Primary Care and Public Health, School of Public Health, Imperial College London, London, United Kingdom, W6 8RP

Table S1. PRISMA checklist

Section and Topic	Item #	Checklist item	Location where item is reported
**TITLE**			
Title	1	Identify the report as a systematic review.	Title page
**ABSTRACT**			
Abstract	2	See the PRISMA 2020 for Abstracts checklist.	1
**INTRODUCTION**			
Rationale	3	Describe the rationale for the review in the context of existing knowledge.	2-3
Objectives	4	Provide an explicit statement of the objective(s) or question(s) the review addresses.	4
**METHODS**			
Eligibility criteria	5	Specify the inclusion and exclusion criteria for the review and how studies were grouped for the syntheses.	5-6
Information sources	6	Specify all databases, registers, websites, organisations, reference lists and other sources searched or consulted to identify studies. Specify the date when each source was last searched or consulted.	5
Search strategy	7	Present the full search strategies for all databases, registers and websites, including any filters and limits used.	4-5, Table S2
Selection process	8	Specify the methods used to decide whether a study met the inclusion criteria of the review, including how many reviewers screened each record and each report retrieved, whether they worked independently, and if applicable, details of automation tools used in the process.	7
Data collection process	9	Specify the methods used to collect data from reports, including how many reviewers collected data from each report, whether they worked independently, any processes for obtaining or confirming data from study investigators, and if applicable, details of automation tools used in the process.	7
Data items	10a	List and define all outcomes for which data were sought. Specify whether all results that were compatible with each outcome domain in each study were sought (e.g. for all measures, time points, analyses), and if not, the methods used to decide which results to collect.	Textbox 1
	10b	List and define all other variables for which data were sought (e.g. participant and intervention characteristics, funding sources). Describe any assumptions made about any missing or unclear information.	Textbox 1
Study risk of bias assessment	11	Specify the methods used to assess risk of bias in the included studies, including details of the tool(s) used, how many reviewers assessed each study and whether they worked independently, and if applicable, details of automation tools used in the process.	8
Effect measures	12	Specify for each outcome the effect measure(s) (e.g. risk ratio, mean difference) used in the synthesis or presentation of results.	N/A
Synthesis methods	13a	Describe the processes used to decide which studies were eligible for each synthesis (e.g. tabulating the study intervention characteristics and comparing against the planned groups for each synthesis (item #5)).	8-9
	13b	Describe any methods required to prepare the data for presentation or synthesis, such as handling of missing summary statistics, or data conversions.	N/A
	13c	Describe any methods used to tabulate or visually display results of individual studies and syntheses.	8-9
	13d	Describe any methods used to synthesize results and provide a rationale for the choice(s). If -analysis was performed, describe the model(s), method(s) to identify the presence and extent of statistical heterogeneity, and software	8-9

Section and Topic	Ite m #	Checklist item	Location where item is reported
		package(s) used.	
	13e	Describe any methods used to explore possible causes of heterogeneity among study results (e.g. subgroup analysis, meta-regression).	7,8
	13f	Describe any sensitivity analyses conducted to assess robustness of the synthesized results.	N/A
Reporting bias assessment	14	Describe any methods used to assess risk of bias due to missing results in a synthesis (arising from reporting biases).	8
Certainty assessment	15	Describe any methods used to assess certainty (or confidence) in the body of evidence for an outcome.	7,8
**RESULTS**			
Study selection	16a	Describe the results of the search and selection process, from the number of records identified in the search to the number of studies included in the review, ideally using a flow diagram.	9, Fig 1
	16b	Cite studies that might appear to meet the inclusion criteria, but which were excluded, and explain why they were excluded.	Fig 1
Study characteristics	17	Cite each included study and present its characteristics.	10-12, Table 2
Risk of bias in studies	18	Present assessments of risk of bias for each included study.	16-17, Table 4, Table S3
Results of individual studies	19	For all outcomes, present, for each study: (a) summary statistics for each group (where appropriate) and (b) an effect estimate and its precision (e.g. confidence/credible interval), ideally using structured tables or plots.	12-16
Results of syntheses	20a	For each synthesis, briefly summarise the characteristics and risk of bias among contributing studies.	19-20
	20b	Present results of all statistical syntheses conducted. If meta-analysis was done, present for each the summary estimate and its precision (e.g. confidence/credible interval) and measures of statistical heterogeneity. If comparing groups, describe the direction of the effect.	20
	20c	Present results of all investigations of possible causes of heterogeneity among study results.	N/A
	20d	Present results of all sensitivity analyses conducted to assess the robustness of the synthesized results.	N/A
Reporting biases	21	Present assessments of risk of bias due to missing results (arising from reporting biases) for each synthesis assessed.	N/A
Certainty of evidence	22	Present assessments of certainty (or confidence) in the body of evidence for each outcome assessed.	N/A
**DISCUSSION**			
Discussion	23a	Provide a general interpretation of the results in the context of other evidence.	18-21
	23b	Discuss any limitations of the evidence included in the review.	18-21
	23c	Discuss any limitations of the review processes used.	19-20
	23d	Discuss implications of the results for practice, policy, and future research.	20-21
**OTHER INFORMATION**			
Registration and protocol	24a	Provide registration information for the review, including register name and registration number, or state that the review was not registered.	Abstract
	24b	Indicate where the review protocol can be accessed, or state that a protocol was not prepared.	3, 5, 7
	24c	Describe and explain any amendments to information provided at registration or in the protocol.	5, 8

Section and Topic	Ite m #	Checklist item	Location where item is reported
Support	25	Describe sources of financial or non-financial support for the review, and the role of the funders or sponsors in the review.	Title page
Competing interests	26	Declare any competing interests of review authors.	Title page
Availability of data, code and other materials	27	Report which of the following are publicly available and where they can be found: template data collection forms; data extracted from included studies; data used for all analyses; analytic code; any other materials used in the review.	22

Table S2. Search strings and records

Table S2. Search strings and records

Database	Search string	References[a]
Embase	(web-based intervention/ OR internet/ OR telemedicine/ OR telehealth/ OR videoconferencing/ OR internet.ab,kw,ti. OR web.ab,kw,ti. OR online.ab,kw,ti. OR remote.ab,kw,ti. OR digital.ab,kw,ti. OR video*.ab,kw,ti. OR virtual.ab,kw,ti. OR technolog*.ab,kw,ti.) AND (group therapy/ OR peer support/ OR group process/ OR (group* adj3 (based OR treatment* OR therap* OR virtual or session* OR peer OR support*)).ab,kw,ti. OR group intervention.ab,kw,ti. OR group*.ti.) AND (exp obesity/ OR obesity management/ OR (obesity or obese).ab,kw,ti. OR specialist weight management.ab,kw,ti. OR Tier 3 weight management.ab,kw,ti. OR (BMI adj1 "35").ab,kw,ti. OR (BMI adj1 "40").ab,kw,ti. OR (BMI adj1 "45").ab,kw,ti.)	(886) 1050<br><br>1341 (31 May 2024)
Medline	(web-based intervention/ OR internet/ OR telemedicine/ OR videoconferencing/ OR internet.ab,kw,ti. OR web.ab,kw,ti. OR online.ab,kw,ti. OR remote.ab,kw,ti. OR digital.ab,kw,ti. OR video*.ab,kw,ti. OR virtual.ab,kw,ti. OR technolog*.ab,kw,ti.) AND (Psychotherapy, Group/ OR peer support/ OR group process/ OR (group* adj3 (based OR treatment* OR therap* OR virtual or session* OR peer OR support*)).ab,kw,ti. OR group intervention.ab,kw,ti. OR group*.ti.) AND (exp obesity/ OR obesity management/ OR (obesity or obese).ab,kw,ti. OR specialist weight management.ab,kw,ti. OR Tier 3 weight management.ab,kw,ti. OR (BMI adj1 "35").ab,kw,ti. OR (BMI adj1 "40").ab,kw,ti. OR (BMI adj1 "45").ab,kw,ti.)	(494) 583<br><br>685 (31 May 2024)
CINAHL	((MH "Internet-Based Intervention") OR (MH "Internet") OR (MH "telemedicine") OR (MH "videoconferencing") OR TI (internet or web or online or remote or digital or video* or virtual or technolog*) OR AB (internet or web or online or remote or digital or video* or virtual or technolog*) OR SU (internet or web or online or remote or digital or video* or virtual or technolog*)) AND ((MH "Psychotherapy, Group") OR (MH "Peer Group" OR MH "Group Processes") OR TI (group* N3 (based or treatment* or therap* or virtual or session* or peer* or support*)) OR AB (group* N3 (based or treatment* or therap* or virtual or session* or peer* or support*)) OR SU (group* N2 (based or treatment* or therap* or virtual or session* or peer* or support*)) TI group* OR AB "group intervention" OR SU group*) AND ((MH "Obesity+") OR TI (obesity or obese) OR	(554) 621<br><br>648 (31 May 2024)

	AB (obesity or obese) OR SU (obesity or obese) OR TI "specialist weight management" OR AB "specialist weight management" OR SU "specialist weight management" OR TI "Tier 3 weight management" OR AB "Tier 3 weight management" OR SU "Tier 3 weight management" OR TI (BMI N1 (35 OR 40 OR 45)) OR AB (BMI N1 (35 OR 40 OR 45)) OR SU (BMI N1 (35 OR 40 OR 45)))	
Web of Science	TS=(internet or web or online or remote or digital or video* or virtual or technolog*) AND TS=((group* Near/3 (based or treatment* or therap* or virtual or session* or peer* or support*)) OR "group intervention") AND TS=(obesity or obese OR "specialist weight management" OR "Tier 3 weight management" OR (BMI Near/1 (35 OR 40 OR 45)))	(557) 638<br><br>845 (31 May 2024)
Cochrane Central[b]	(MeSH descriptor:[Internet-Based Intervention] explode all trees OR MeSH descriptor:[Internet] explode all trees OR MeSH descriptor:[Telemedicine] explode all trees OR MeSH descriptor:[Videoconferencing] explode all trees OR (internet or web or online or remote or digital or video* or virtual or technolog*):ti,ab,kw (Word variations have been searched)) AND (MeSH descriptor:[Group Processes] explode all trees OR MeSH descriptor:[Peer Group] explode all trees OR MeSH descriptor:[Psychotherapy, Group] explode all trees OR (group* Near/3 (based or treatment* or therap* or virtual or session* or peer* or support*)):ti,ab,kw (Word variations have been searched) OR (group*):ti (Word variations have been searched) OR ("group intervention"):ab (Word variations have been searched)) AND (MeSH descriptor:[Obesity] explode all trees OR MeSH descriptor:[Obesity Management] explode all trees OR (obesity or obese OR "specialist weight management" OR "Tier 3 weight management"):ti,ab,kw OR (BMI Near/1 (35 OR 40 OR 45)):ti,ab,kw (Word variations have been searched)) NOT (embase OR pubmed):an	(255)<br><br>409 (31 May 2024)
Total		(2736) 3137 3928 (31 May 2024)
Total	*(with (1024) 1183 duplicates removed by EndNote)*<br><br>*With 3,293 duplicates (including those from previous searches) removed*	(1712) 1954<br><br>635 (31 May 2024)

[a]Reference numbers in brackets refer to the original search results retrieved in March 2021. Due to a delay in the systematic review execution, the searches were re-run in April 2022; these were the totals used in the search and screening for this review.

Table S3. Data extraction, risk of bias, and quality assessment table

**See attached excel document*
